# Editorial: Advances in chemotherapy-resistant hepatocellular carcinoma

**DOI:** 10.3389/fmed.2023.1325304

**Published:** 2023-11-13

**Authors:** Shui Liu, Qiao Li, Yongzhi Li, Jiyao Sheng

**Affiliations:** ^1^Department of Hepatobiliary and Pancreatic Surgery, The Second Hospital of Jilin University, Jilin University, Changchun, China; ^2^Jilin Engineering Laboratory for Translational Medicine of Hepatobiliary and Pancreatic Diseases, Changchun, China

**Keywords:** hepatocellular carcinoma, cholangiocarcinoma, chemotherapy, molecular-targeted therapy, drug resistance mechanisms, combined chemotherapy

Primary liver cancer, including hepatocellular carcinoma and cholangiocarcinoma, has a poor prognosis. According to the American Cancer Society statistics, the 5-year survival rate of liver cancer is < 20%. Chemotherapy is the preferred method to kill residual cancer cells after surgery and prolong the survival time of inoperable patients, but most cases are insensitive to chemotherapeutic agents, which leads to poor efficacy and restricts the widespread clinical application of chemotherapy in liver cancers. The reason is that liver cancer cells have primary resistance to chemotherapy drugs or acquired resistance in the process of treatment.

In recent years, the mechanism of chemotherapy resistance to liver cancer has gradually become clear with more and more studies on it, and new combined chemotherapy schemes have also been emerging. These have brought new hope for reversing the chemotherapy resistance of liver cancer. Therefore, this topic will focus on the related content of reversing chemotherapy of primary liver cancer and face the clinical research and basic research of primary liver cancer chemotherapy.

The Research Topic consists of four original research papers, two reviews, and one case report from prominent researchers in the field and provides readers of the journal with advanced findings in combined therapy of liver cancer, mechanisms of drug resistance, and corresponding possible strategies.

Tyrosine kinase inhibitor (TKI) can inhibit tyrosine kinase activity to interfere with the growth and reproduction of liver cancer cells (1). TKIs have currently become one of the main drugs for molecular-targeted therapy of liver cancer since the introduction of the first TKI Sorafenib (2, 3). The drug resistance of multi-kinase inhibitors limits their clinical application (4). One study by Jiang et al. focused on the drug resistance mechanism of kinase inhibitors in the treatment of hepatocellular carcinoma. In recent years, multi-target kinase inhibitors for HCC, such as sorafenib, Lenvatinib (LVN), cabozantinib, and regorafenib, have shown promising prospects in the treatment of HCC and produced considerable benefits in clinical. The development of drug resistance becomes the major obstacle. Let and fellows mainly reviewed the mechanisms of sorafenib resistance in HCC from two aspects: primary drug resistance including mutation of EGFR and enrichment of CSC, and acquired drug resistance including compensatory activation of the PI3K/Akt pathway. Compensatory activation of the MAPK/ERK pathway, epithelial-mesenchymal transition (EMT), metabolic reprogramming, autophagy, non-coding RNAs, apoptosis resistance and deregulated cell cycle control, and summarized the mechanisms of other kinase inhibitors (Figure 1). They also discussed possible strategies based on the mechanisms to improve the treatment outcome of HCC patients.

**Figure 1:**
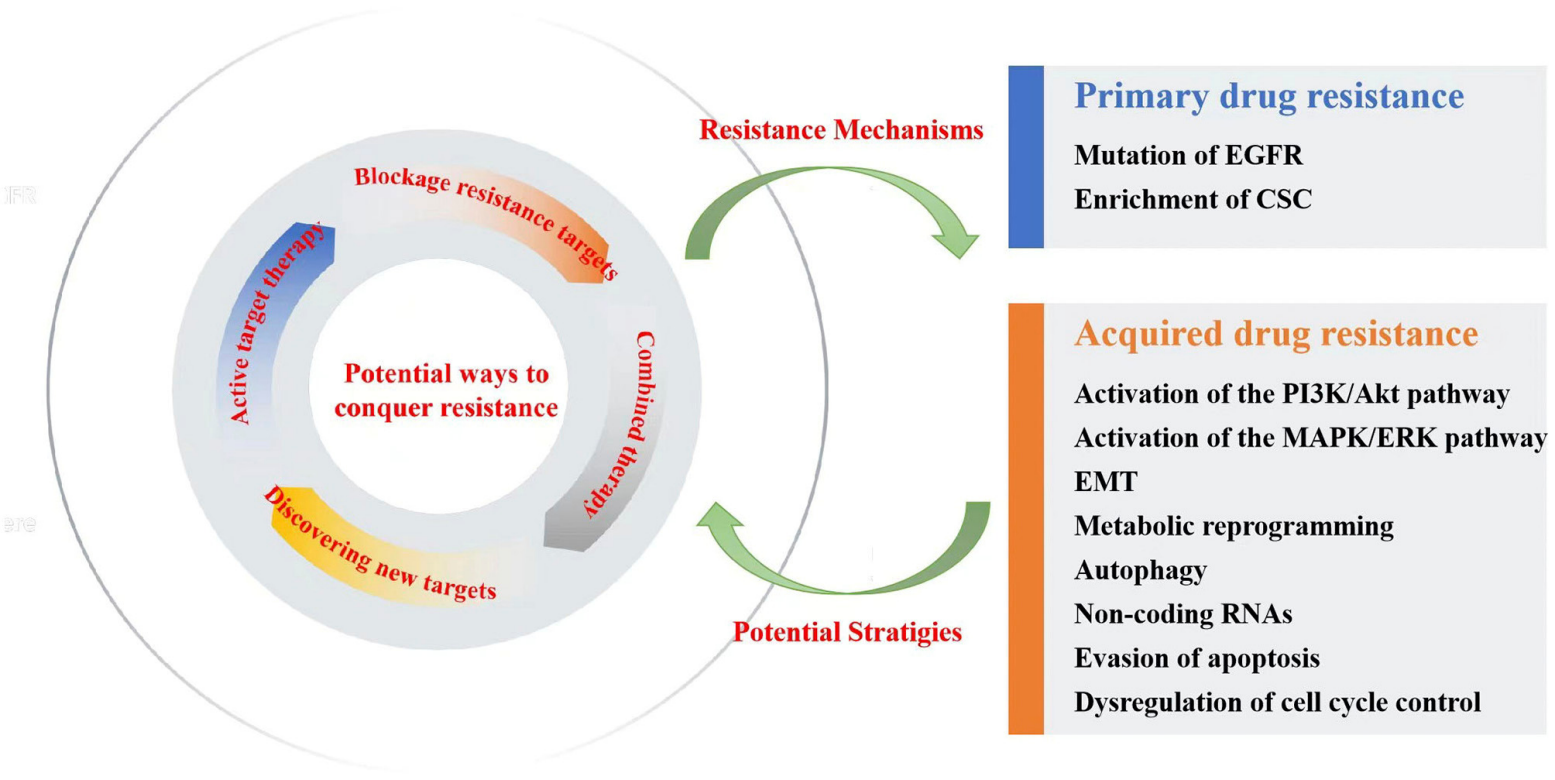
Resistance mechanisms and potential ways to conquer liver cancer with Sorafenib.

A secondary study by Bo and Chen mainly focused on the mechanisms of LVN resistance and potential ways to overcome it. The resistance mechanisms in the paper included EMT-related resistance, DNA damage response (DDR)-involved resistance, ferroptosis-participated resistance, autophagy-related resistance, RNA involved in regulating resistance, RNA modification-jointed resistance, cytokine overexpression-related resistance, and post-translational modifications attended in resistance. Subsequently, four potential ways to conquer liver cancer were summarized, which included active target therapy to enhance efficacy and prolong the time to the onset of resistance, combined therapy to promote sensitization of LVN, blockage of the resistance targets, and using new technologies to discover new resistance-conferred target.

Cholangiocarcinoma (CCA) has obvious primary multidrug resistance and is generally resistant to cisplatin and other chemotherapy drugs (5). High glycolytic levels may be associated with chemotherapy resistance to cholangiocarcinoma (6). Another study by Qin et al. explored the role of metabolic reprogramming in cisplatin resistance of CCA. In this study, dichloroacetate (DCA), which was a specific inhibitor of PDK, was confirmed to change the metabolic model from glycolysis to aerobic oxidation after cisplatin treatment, and the metabolic reprogramming increased mitochondrial reactive oxygen species levels, which promoted cell cycle arrest, increased the expression of antioxidant genes, and activated autophagy via cell apoptosis, cell cycle, and mito-TEMPO. The inhibition of autophagy could increase the synergistic effect of DCA and cisplatin.

Another study by Casadei-Gardini et al. presented survival trends over almost 20 years in 922 patients with advanced CCA in Italy, who were treated with systemic chemotherapy. The study included 14 centers within the Cholangiocarcinoma Italy Group Onlus. The progression-free survival (PFS) of patients at first-line therapy was 13.90 and 7.07% at 12 and 18 months, respectively; overall survival (OS) was 43.90 and 26.00% at 12 and 18 months, respectively. The PFS of patients at second-line therapy was 5.94 and 2.83% at 12 and 18 months, respectively; OS was 29.60 and 17.80% at 12 and 18 months. The study confirmed that modest overall advances could be achieved with first- and second-line chemotherapy in advanced CCA.

Another study by Hu, Yang et al. reviewed data from 1,251 consecutive hepatocellular carcinoma patients who underwent liver resection at the Sun Yat-Sen University Cancer Center. The patients were divided into two groups: patients who received liver resection alone (LR group), and patients who were treated with FOLFOX-HAIC followed by liver resection (HLR group), and the propensity score matching (PSM) was conducted between the two groups. Then, multivariable Cox regression analysis and nomogram were performed. After PSM, according to the initial tumor characteristics, the 1-, 2-, and 3-year overall survival rates were 85.4, 72.0, and 67.2% in the LR group and 95.2, 84.7, and 75.9% in the HLR group, respectively (*p* = 0.014). After PSM, according to the preoperative tumor characteristics, the 1-, 2-, and 3-year OS rates were 87.9, 76.6, and 72.3% in the LR group and 95.4, 84.4, and 75.1% in the HLR group, respectively (*p* = 0.24). Preoperative FOLFOX-HAIC is confirmed to be associated with a longer survival outcome for HCC patients.

Hu, Yang et al. also performed a retrospective study including 120 resectable HCC patients with portal vein tumor thrombus (PVTT) at Sun Yat-sen University Cancer Center. The patients were divided into two groups: patients who received hepatectomy alone (Surgery group) and patients who received neoadjuvant hepatic arterial infusion chemotherapy (HAIC) followed by hepatectomy (HAIC-Surgery group). Logistic regression analysis was conducted to develop a model predicting the response to neoadjuvant HAIC. The OS rates for the HAIC-Surgery group at 1, 3, and 5 years were 94.9, 78, and 66.4%, respectively, compared with 84.6, 47.6, and 37.2% in the Surgery group (*p* < 0.001). The RFS rates were 88.7, 56.2, and 38.6% vs. 84.9, 38.3, and 22.6% (*p* = 0.002). Neoadjuvant HAIC followed by hepatectomy is associated with a longer survival outcome than hepatectomy alone for HCC patients with PVTT and the survival benefit depends on patients' response to neoadjuvant FOLFOXHAIC.

Combined hepatocellular-cholangiocarcinoma (cHCC-CCA) is characterized by high invasiveness and poor prognosis (7, 8). Optimal treatment is important to improve the prognosis of cHCC-CCA patients. Another study by Zhou et al. reported a case of a patient with postoperative metastatic chemotherapy-resistant cHCC-CCA who exhibited a durable response and reasonable tolerability to a combination therapy consisting of the anti-PD1 having a low tumor mutational burden (TMB-L), microsatellite stability (MSS), and negative programmed cell death 1 ligand 1(PD-L1). The combination regimen of immune checkpoint inhibitor sintilimab, multi-kinase inhibitor Lenvatinib, and chemotherapy with nab-paclitaxel, which targets both the HCC and ICC components, may represent a promising treatment option for patients with cHCC-CCA.

Liver cancer is a common malignant tumor and the third leading cause of cancer-related deaths worldwide (9). Surgical resection is currently the most effective curative treatment for liver cancer (10, 11). However, most patients with liver cancer are at the advanced stage when diagnosed, and surgery treatment is not available for more than 50% of them (12, 13). Chemotherapy and molecular-targeted therapy have become one of the important options for the treatment of liver cancer, but drug resistance limited their clinical application (14, 15). This Research Topic focused on the clinical application and drug-resistance mechanism of chemotherapy drugs and molecular-targeted drugs, including clinical research, basic research, and review, and explored the drug-resistance mechanisms of liver cancer and possible solutions from multiple perspectives. The Research Topic provides new possibilities for exploration and research to achieve better prognosis of liver cancer patients.

## Author contributions

SL: Writing—original draft. QL: Writing—review & editing. YL: Writing—review & editing. JS: Writing—review & editing.
